# Histopathological Alterations and Dysregulation of Type I Interferon Signaling in the Human Cerebral Cortex During Severe Dengue

**DOI:** 10.1002/jmv.70644

**Published:** 2025-10-14

**Authors:** Leandro Mantovani de Castro, Elaine Raniero Fernandes, Juarez Antonio Simoes Quaresma, Carmen Lucia Penteado Lancelotti, Maria Irma Seixas Duarte, Leda Viegas de Carvalho, Ricardo Penny, Pedro Fernando da Costa Vasconcelos, Evandro Sobroza de Mello, Mirian Nacagami Sotto, Carla Pagliari

**Affiliations:** ^1^ Instituto de Biociências da Unesp São Vicente São Paulo Brasil; ^2^ Programa de Pós‐graduação em Ciências da Saúde‐ IAMSPE São Paulo Brasil; ^3^ Departamento de Patologia, Faculdade de Medicina Universidade de São Paulo São Paulo Brasil; ^4^ Instituto Pasteur Secretaria de Estado da Saúde São Paulo Brasil; ^5^ INCT‐VER Universidade do Estado do Pará Pará Brasil; ^6^ Faculdade de Ciências Médicas da Santa Casa de São Paulo Serviço de Anatomia Patológica São Paulo Brasil; ^7^ Serviço de Anatomia Patológica – Hospital Garcia de Orta EPE, ULS Almada Seixal Portugal; ^8^ Hospital Guilherme Álvaro, Serviço de Verificação de Óbito Santos São Paulo Brasil

**Keywords:** CNS, histopathology, IRF2, RIG‐I, severe dengue, STING

## Abstract

Dengue virus (DENV) is a major arthropod‐borne pathogen, endemic in over 100 countries and posing global health challenges. While innate immune responses and viral evasion mechanisms have been extensively studied in animal models and mononuclear cells, severe dengue can affect multiple tissues, including the central nervous system (CNS), leading to neurological manifestations. However, the CNS immune response remains poorly understood. This study analyzed molecules linked to innate immunity in CNS lesions from fatal dengue cases. Histopathological examination of the cerebral cortex revealed marked neuronal damage—chromatolysis, pyknotic nuclei—accompanied by microglial hyperplasia, white matter demyelination, perivascular inflammation, vascular congestion, vasogenic edema, and occasional hemorrhage or meningitis. DENV antigen was detected in endothelial cells of cortical and leptomeningeal vessels and in glial cells or macrophages. Immunohistochemistry revealed altered expression of innate immune markers: RIG‐I was sparsely expressed, STING was absent, and IFN‐α/β levels were reduced compared to controls. Notably, IRF2 expression was markedly elevated, with strong labeling in neurons, glial, and endothelial cells. These findings suggest an atypical pattern of immune activation in the CNS during severe dengue and highlight a potential role for IRF2 in modulating cerebral immune responses, offering new insights into the neuropathogenesis of dengue.

## 1 Introduction

Dengue is an arboviral disease caused by an enveloped RNA virus belonging to the Flaviviridae family and the *Flavivirus* genus, that affects millions of people worldwide each year. Present in over 100 countries, it represents one of the greatest challenges to healthcare systems [1]. Brazil has experienced a significant increase in the number of dengue cases, with millions of reported and epidemics of the disease tend to occur in cycles, with changes in the predominant serotypes [2, 3]. DENV is known to circulate worldwide in four distinct serotypes: DENV‐1, DENV‐2, DENV‐3, and DENV‐4. Infection with any of these serotypes can cause dengue fever (DF) within 2 to 7 days following the bite of an infected mosquito. In some cases, the illness may rapidly evolve into dengue hemorrhagic fever (DHF), and if not properly diagnosed or managed, it can progress to dengue shock syndrome (DSS) [4, 5].

The pathogenesis of dengue is complex, multifactorial, and the mechanisms are not yet fully understood. It has been shown an intricate interaction between viral, immunological, and host factors [6, 7]. This interaction includes an increase in antibody‐mediated immune response in secondary dengue infections, leading to enhanced capture of virus‐antibody complexes by mononuclear phagocytes [8, 9, 10]. Additionally, studies have also shown the involvement of the release of a cytokine storm by immune system cells [11, 12], a lymphocyte hyperactivation [13], and the influence of the specific viral serotype causing the infection [14, 15].

In addition to immune cells, DENV has been detected in other types of cells, accessing, and compromising the homeostasis of a variety of organs in the body, especially in cases that progress to DHF or DSS. One of these areas of compromise is the central nervous system, with neurological clinical manifestations observed with an incidence rating varying from 0.5% to 20% [16, 17]. Based on the pathogenesis, the neurological complications found in patients with dengue virus infection can be classified into three categories: (a) those related to metabolic disturbance, such as encephalopathy; (b) those characterized by viral invasion, such as encephalitis, meningitis, myositis, and myelitis; and (c) those caused by the development of autoimmune reactions, including antibody‐dependent enhancement (ADE), Guillain‐Barré syndrome (GBS), optic neuritis, and myelitis [17, 18].

The infectivity of DENV and the mechanisms of immune response in the CNS have been primarily explored in animal models and human cell lines [19, 20, 21, 22, 23], showing that the presence of the blood‐brain barrier, with brain microvascular endothelial cells associated to pericytes, astrocytes, and microglia play an important role in the neuropathogenesis and may be distinct of other organs affected in cases of severe dengue [24, 25, 26, 27].

A recent study with post‐mortem brain histopathological samples provided information about the neurotropism of DENV and the inflammatory response in the context of severe disease. It was found that DENV can access the brain parenchyma and replicates in endothelial cells, neurons, and microglia. Some pro‐inflammatory mediators, such as TNF‐α, IFN‐γ, NO, and HMGB1 (high mobility group box), were observed [28].

Innate immunity plays an important role in quickly controlling the replication of RNA viruses by limiting the spread of the virus through the detection of pathogen associated molecular patterns (PAMPs) by pattern recognition receptors (PRRs), activating responses such as the production of pro‐inflammatory cytokines [7, 29]. The cytoplasmic retinoic acid‐inducible gene I (RIG‐I), melanoma differentiation‐associated protein 5 (MDA5), along with endosomal Toll‐like receptor 3 (TLR3) and TLR7, are the main PRRs associated with the recognition of flaviviruses, particularly in DENV infection [30, 31].

Beyond RNA sensors like RIG‐I, the STING (Stimulator of Interferon Genes) pathway contributes significantly to the induction of type I interferons (IFN‐α/β), which are key antiviral cytokines produced early in response to pathogen recognition [32, 33]. Although STING is classically activated by cytosolic DNA via cGAS, mitochondrial damage during DENV infection may lead to DNA release and subsequent pathway activation. Once activated, STING triggers signaling cascades resulting in ISG expression and IFN‐I production [34, 35].

Interferon regulatory factors (IRFs) are a family of transcription factors that play a central role in coordinating antiviral responses, particularly by regulating type I interferon signaling and the expression of interferon‐stimulated genes (ISGs). Among them, IRF‐2 stands out for its dual role as both a transcriptional regulator and repressor, modulating IRF‐1‐driven gene expression. By fine‐tuning these pathways, IRF‐2 helps maintain immune homeostasis and protects host tissues from excessive immune activation [36, 37].

Considering the increase in the number of dengue cases with neurological clinical manifestations, the lack of information on DENV in human nervous tissue and recent findings in animal experimental models and other organs, the present study evaluated, through immunohistochemistry, the expression of a series of markers related to the innate immune response in autopsies of patients diagnosed with severe dengue, to understand cellular and molecular aspects related to the disease.

## 2 Materials and Methods

### 2.1 Cases

In this study, eight cerebral cortex lesions from autopsies of patients previously diagnosed with severe dengue were retrospectively analyzed. These specimens were obtained from the Death Verification Service of Guilherme Alvaro Hospital, Santos, Brazil. The selection of cases was based on previous clinical and serological data and/or identification of specific antigens shown through immunohistochemical reactions. For comparative analysis, five brain fragments obtained during routine autopsies were used as negative controls. These fragments were from patients at the Death Verification Service of the Clinical Hospital, Medical School, University of Sao Paulo. This control group was selected based on cause of death and included individuals who died from diseases not involving the CNS and had no report of an autoimmune disease. The research was approved by the Ethics and Research Committee of Medical School, University of Sao Paulo (Protocol number: 253/12), in accordance with the National Health Council (through Resolution No. 466/12) and the Declaration of Helsinki.

### 2.2 Serological Detection of Dengue

Serum samples were tested for anti‐dengue virus IgM and IgG antibodies using a commercial enzyme‐linked immunosorbent assay (ELISA) kit, according to the manufacturer's instructions. Briefly, patient sera were added to microplate wells precoated with dengue antigens, followed by incubation with enzyme‐labeled anti‐human IgM or IgG conjugates. After substrate addition, optical density (OD) was measured at 450 nm. Results were interpreted based on the ratio between the sample OD and the cut‐off value: samples with index values below 0.8 were considered nonreactive, those above 1.2 reactive, and values between 0.8 and 1.2 were classified as indeterminate. All assays were performed in duplicate.

### 2.3 Histopathological Analysis

The fragments from cerebral cortex were fixed in 10% buffered formalin, dehydrated in ethanol, clarified in xylene and blocked in paraffin. In sequence, each sample was sectioned in 4 µm‐thick units, deparaffinized into xylene and rehydrated with alcohol series (100%, 90%, 80% and 70%). The histological sections were stained with hematoxylin and eosin (HE) and visualized under a light microscopy (BEL Engineering, Monza, Italy). Digital images obtained using Capture 2.1 software.

### 2.4 Immunohistochemistry

Histological slides were dewaxed, hydrated, and incubated in peroxide 3% solution for 30 min at room temperature, washed in running water for 5 min and equilibrated in PBS buffer. Then, slides were incubated for 20 min in a water bath at 95°C in TRIS‐EDTA buffer solution (pH 9.0). The slides were covered with blocking solution (5% skimmed milk powder diluted in PBS) and incubated for 20 min at room temperature. The specific primary antibody (described in Table 1) was added in sufficient quantity to cover the fragments, and incubated overnight in a humid chamber, at 4°C. For the next step, different Horseradish Peroxidase Detection Systems ready to use were applied (Table 1). The reactions were visualized by applying 0.5% DAB diluted in phosphate‐buffered saline (PBS) containing 0.01% hydrogen peroxide (H₂O₂) for 1–2 min, followed by counterstaining with Harris' Haematoxylin for 1 min.

**Table 1:** Primary antibodies and detection systems used in immunohistochemistry.

Antibody	Mark/code	Dilution	Kit used
Polyclonal mouse anti‐dengue virus type II	Evandro Chagas Institute/PA	1:100	Advance, Dako
Polyclonal goat anti‐RIG‐I	Abcam/Ab111037	1:100	Immpress, Vector
Recombinant Monoclonal rabbit anti‐STING	Abcam/Ab227705	1:400	Immpress, Vector
Polyclonal rabbit anti‐IRF2	Proteintech/125251AP	1:100	Ultravision, Thermo
Monoclonal rabbit anti‐IFN‐alpha/beta	Novus/NBP83119	1:200	Ultravision, Thermo

For quantitative analysis of immunostained cells, images were obtained using an optical microscope (BEL Engineering, Monza, Italy) coupled to a microcomputer, and quantification of immunostained cells was performed using Capture 2.1 software. Thirty microscopic fields (×40 objective) from the parenchyma and perivascular region of each histological section for each target antigen were examined. The number of cells per mm² was determined by the average of the immunostained cells in the analyzed areas. Statistical analysis was performed using the GraphPad Prism version 5.0 program (GraphPad Software Inc., San Diego, California, USA), using the Mann–Whitney test with the level of significance set at 95%.

## 3 Results

According to autopsy data, the cases evaluated in this study involved eight patients aged between 26 and 47 years, except for a 4‐year‐old child. These cases rapidly progressed to severe dengue, with involvement of various organs, showing macroscopic changes, primarily in the liver, lungs, and kidneys. In two cases, cerebral edema was also observed during the anatomical pathology examination.

Dengue serology (IgM and IgG) was positive in seven patients. The case with negative serology showed a positive result in immunohistochemical analysis, which revealed the presence of DENV antigens in both liver and brain tissues. This scenario is consistent with an early acute‐phase infection, in which the sample may have been collected before seroconversion, or with a condition where the humoral immune response is delayed or insufficient. In this case, although anti‐dengue IgM and IgG antibodies were undetectable by ELISA, immunohistochemical analysis provided direct evidence of DENV antigens in the affected tissues.

**Figure 1:**
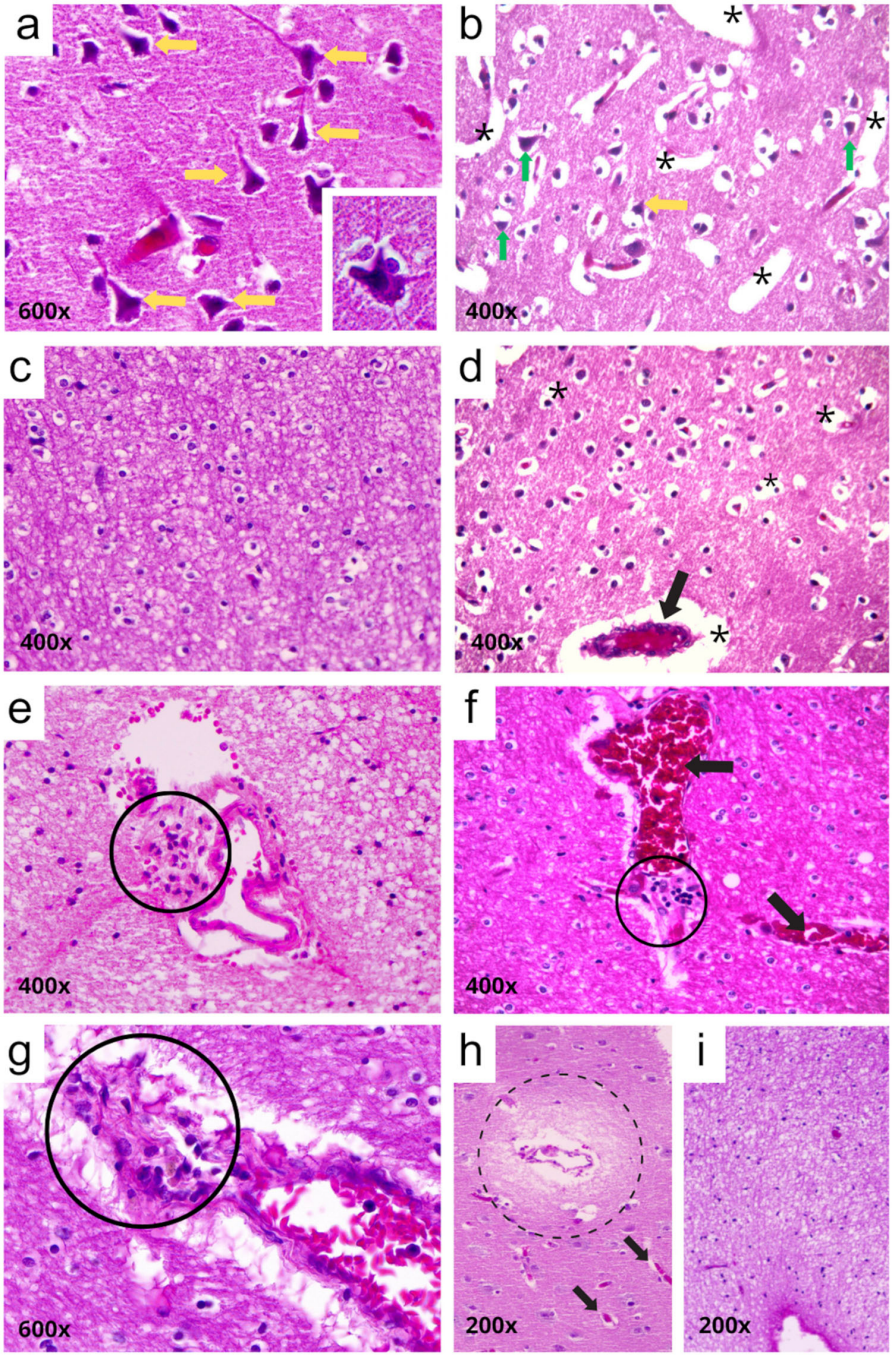
Representative histopathological features of the cerebral cortex in patients with severe dengue. (a) Retracted cortical neurons with pyknotic nuclei and central chromatolysis (yellow arrows, enlarged neuron highlighted in a). (b) Edema (asterisk) and neuronal retraction with pyknotic nuclei (green arrows). (c, d) Microglial hyperplasia and edema (asterisks). (e–g) Perivascular inflammatory infiltrates (solid black circles); congested vessels in (d, f) (black arrows). (h) Vasogenic interstitial edema (dashed black circle). (i) Demyelinated white matter (whitish staining). Magnifications: (a, g) 600×; (b–f) 400×; (h, i) 200×. Staining: Hematoxylin and Eosin.

**Figure 2:**
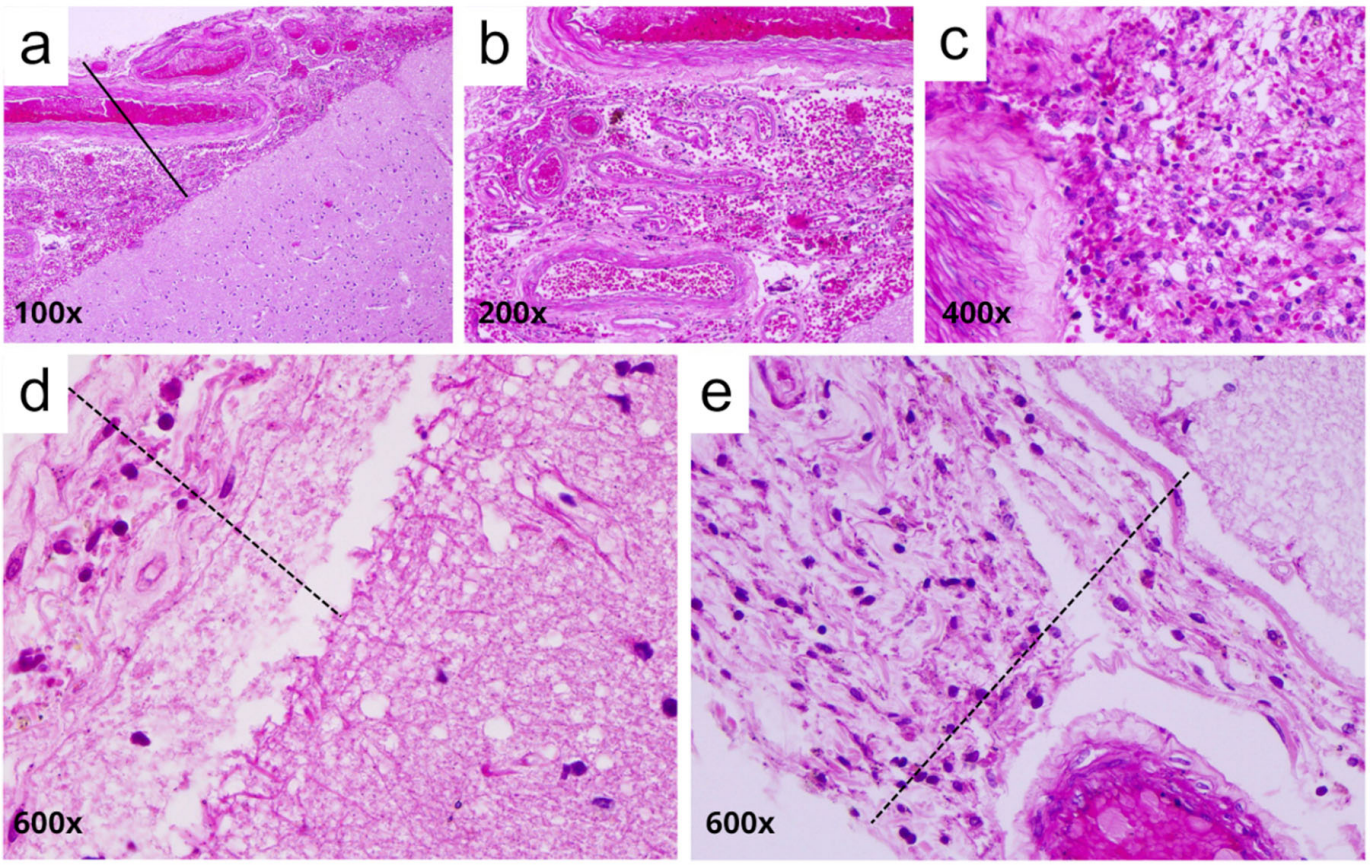
Representative histopathological features of the meninges in patients with severe dengue. (a–c) Hemorrhage; (d, e) meningitis. All images show meningeal thickening (solid line in hemorrhage; dashed line in meningitis). Magnifications: (a) 100×; (b) 200×; (c) 400×; (d, e) 600×. Staining: Hematoxylin and Eosin.

Qualitative histopathological analysis revealed a series of alterations in the cerebral cortex. The most common findings included cortical neurons with contracted cytoplasm and pyknotic nuclei or central chromatolysis, microglial hyperplasia and demyelinated white matter (Table 2, Figure 1a–d). Some cases also presented one or more of the following alterations: perivascular inflammatory infiltrate, vascular congestion, vasogenic interstitial edema and demyelinated white matter (Table 2, Figure 1e–i). In addition, thickening of the leptomeningeal region was observed, associated with hemorrhage or meningitis (Table 2, Figure 2).

**Table 2:** Casuistic and immunohistopathological data.

Patient	Age	Sex	Clinical summary	Dengue serology IGG/IGM	DENV2 IHC (cerebral cortex)	Histopathological (cerebral cortex)
1	4	M	Fever and vomit, progressing within a few hours to cyanosis, hypothermia, and hemodynamic instability, DHF	7.4/1.4	P (+), V (+), M (+)	Cortical neurons contracted with pyknotic nuclei; subcortical white matter demyelinated likely due to hypoxia. Sign of irreversible injury, intense and diffuse vascular congestion. Presence of microglial hyperplasia and perivascular inflammatory infiltrates.
2	30	F	DHF; Sepsis	3.6/3.9	P (+), V (+), M (+)	Cortical neurons contracted with pyknotic nuclei, subcortical white matter demyelinated, microglial hyperplasia
3	28	F	Fever and pain for 3 days, progressing to hypotension and refractory shock despite fluid infusion	4.4/2.2	negative	few neurons with pyknotic nuclei
4	37	M	DHF, splenomegaly	8.8/2.8	P (+), V (+), M (+)	Meningitis; intense and diffuse vascular congestion; Cortical neurons contracted with pyknotic nuclei and central chromatolysis, presence of amylaceous bodies, microglial hyperplasia
5	35	F	Acute respiratory failure; vascular bleeding, Systemic Inflammatory Response Syndrome (SIRS), Disseminated Intravascular Coagulation, DHF	0.4/0.4	P (+), V (+), M (#)	Neurons contracted with pyknotic nuclei and central chromatolysis, microglial hyperplasia and intense and diffuse vascular congestion
6	47	F	Suspected dengue for 4 days, progressing with severe abdominal pain	7.5/2.4	P (+), V (+), M (+)	Meningitis; hemorrhage in the meninges; foci of congestion, neurons with pyknotic nuclei
7	41	F	Persistent fever (1 week), myalgia, nausea, vomiting, and gingival bleeding. Progressed to acute respiratory failure, renal failure, hepatocellular injury, leading to multiple organ failure after 3 days	5.3/2.8	P (+), V (+), M (‐)	neurons with pyknotic nuclei
8	26	M	Widespread body pain, fever and diffuse bruising	R/R (≥ 1.2)*	P(+), V(+), M(+)	Intense hemorrhage in the meninges (perivascular hemorrhage in the subarachnoid space); vascular congestion; vasogenic interstitial edema; contracted neurons with pyknotic nuclei; central chromatolysis.

*Note:* DENV serology values: R=Reactive, ≥ 1.2 index; nonreactive, ≤ 0.8 index. For patients 1–7, serology was performed using a kit that reported results as index values (sample OD/cut‐off). For patient 8, a different kit was employed, which provided only a qualitative result (“Reactive”) without the corresponding index value. According to the laboratory report, this result indicated a reactivity equivalent to an index ≥ 1.2; #: There was no meningeal region in the fragment.

Abbreviations: DHF, dengue hemorrhagic fever; F, female; IHC, Immunohistochemistry; M, male; M, meninges; P, Parenchyma; V, vessels; +, positive.

**Figure 3:**
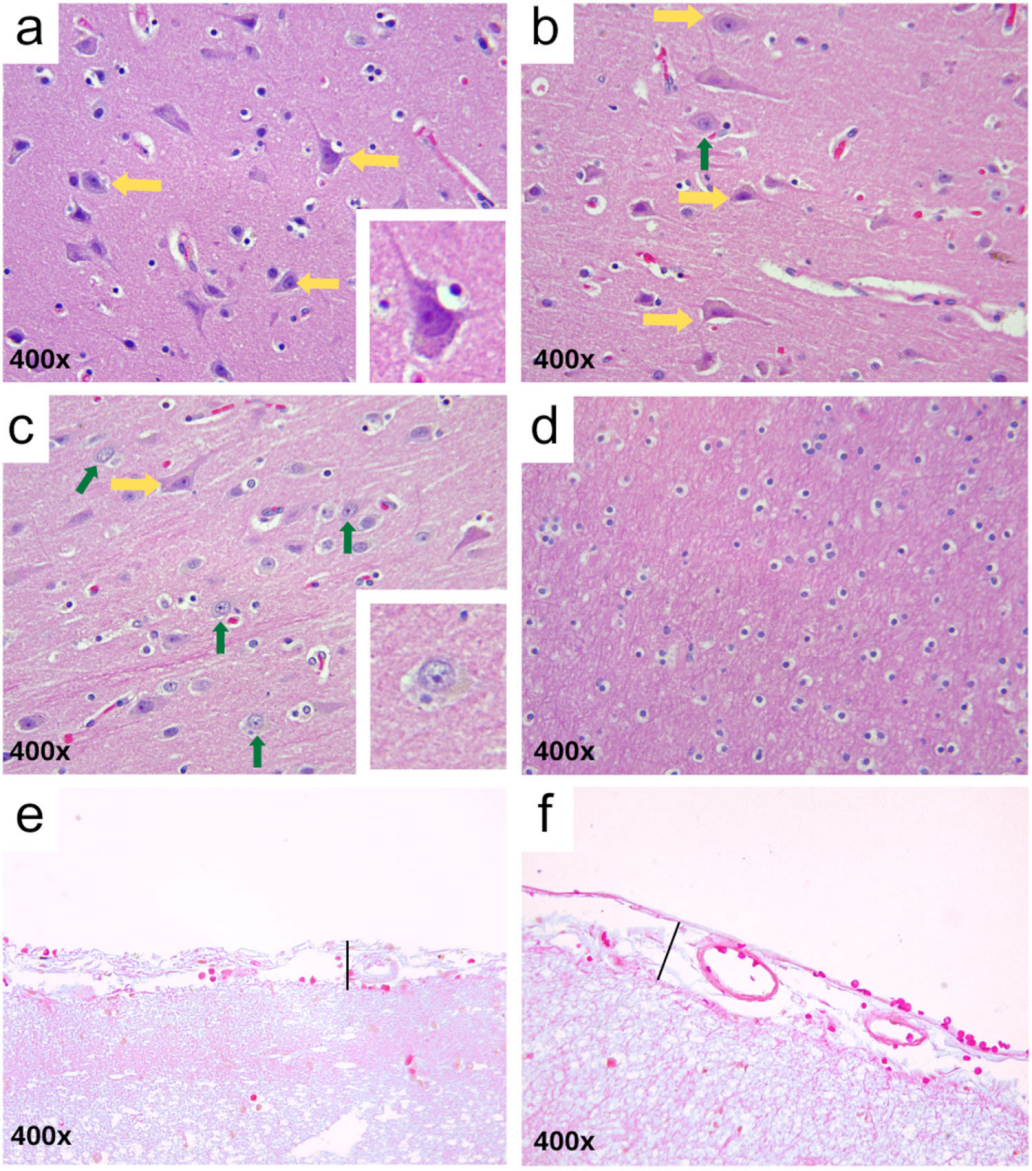
Representative images of the cerebral cortex from control group patients. (a–c) Preserved parenchyma without evidence of edema or neuronal retraction. Most neurons show an intact perikaryon with well‐defined nuclear membrane and prominent nucleolus (yellow arrows; enlarged cell highlighted in a). In (c), some neurons also display fine chromatin and a distinct nucleolus (green arrows, enlarged cell highlighted in c). (d) White matter region with preserved morphology. (e–f) Meningeal region with preserved structure and typical thickness (solid black line). (a–f) 400×. Staining: Hematoxylin and Eosin.

In the control group cases, the parenchyma of both the gray and white matter regions of the cerebral cortex displayed preserved histoarchitecture. Most neuronal cell bodies exhibited well‐defined nuclear membranes and prominent nucleoli. Some nuclei presented dispersed (fine) chromatin, and glial cells were evenly distributed throughout the tissue (Figure 3a–d). The leptomeningeal region showed normal thickness and no apparent alterations (Figure 3e–f).

**Figure 4:**
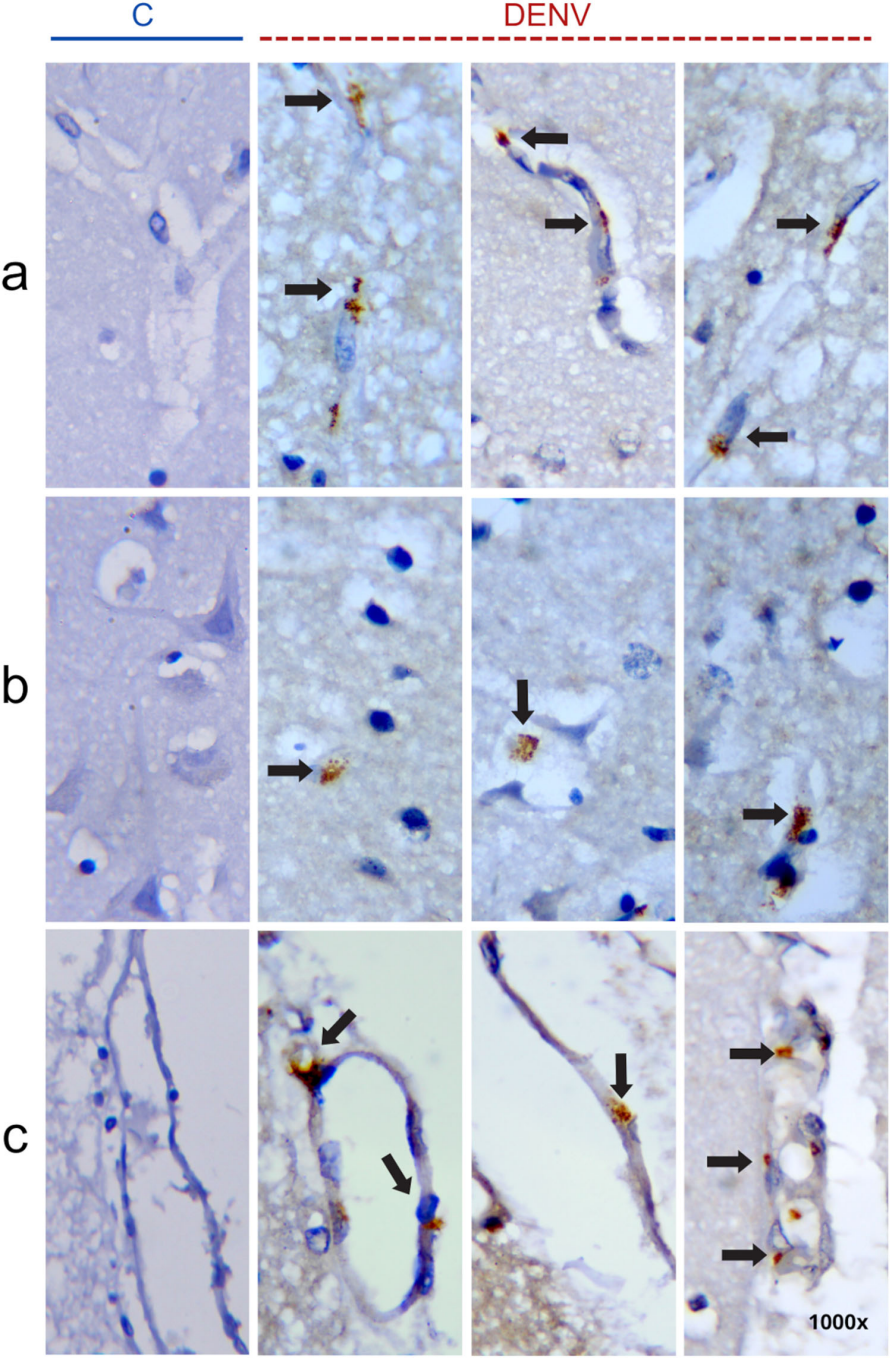
Representative images showing the presence of DENV antigens in brain tissue, detected by immunohistochemistry. (a) Brown‐stained immunopositive cells (black arrows) are observed in blood vessels within both gray and white matter of the cortex. (b) Immunopositive cells are also present in cortical parenchyma. (c) Immunolabeling in the leptomeningeal region. The left column (indicated by a solid blue line) shows control group samples with no detectable DENV antigen immunoreactivity; the remaining columns represent dengue cases (indicated by a red dashed line). All images at 1000× magnification. Staining: immunoperoxidase with Harris hematoxylin counterstain.

The antigen for DENV was detected in the cerebral cortex in 7 cases (Table 2). There was intense cytoplasmic staining, possibly endothelial cells in vessels located in gray and white matter. Endothelial cell nuclei are typically oval or elongated and aligned with the axis of the blood vessel. They display dispersed chromatin, with predominance of euchromatin, and lack the dense heterochromatin network that characterizes microglial cells [38] (Figure 4a, DENV). Additionally, immunostaining was observed in cortical parenchymal cells, suggestive of macrophages or glial cells (Figure 4b, DENV). No evident staining was observed in neuronal cell bodies. This staining pattern was also observed in some cases in the leptomeninges region (Figure 4c, DENV). As expected, any detection was observed in samples of non‐dengue cases (Figure 4a‐c, C).

**Figure 5:**
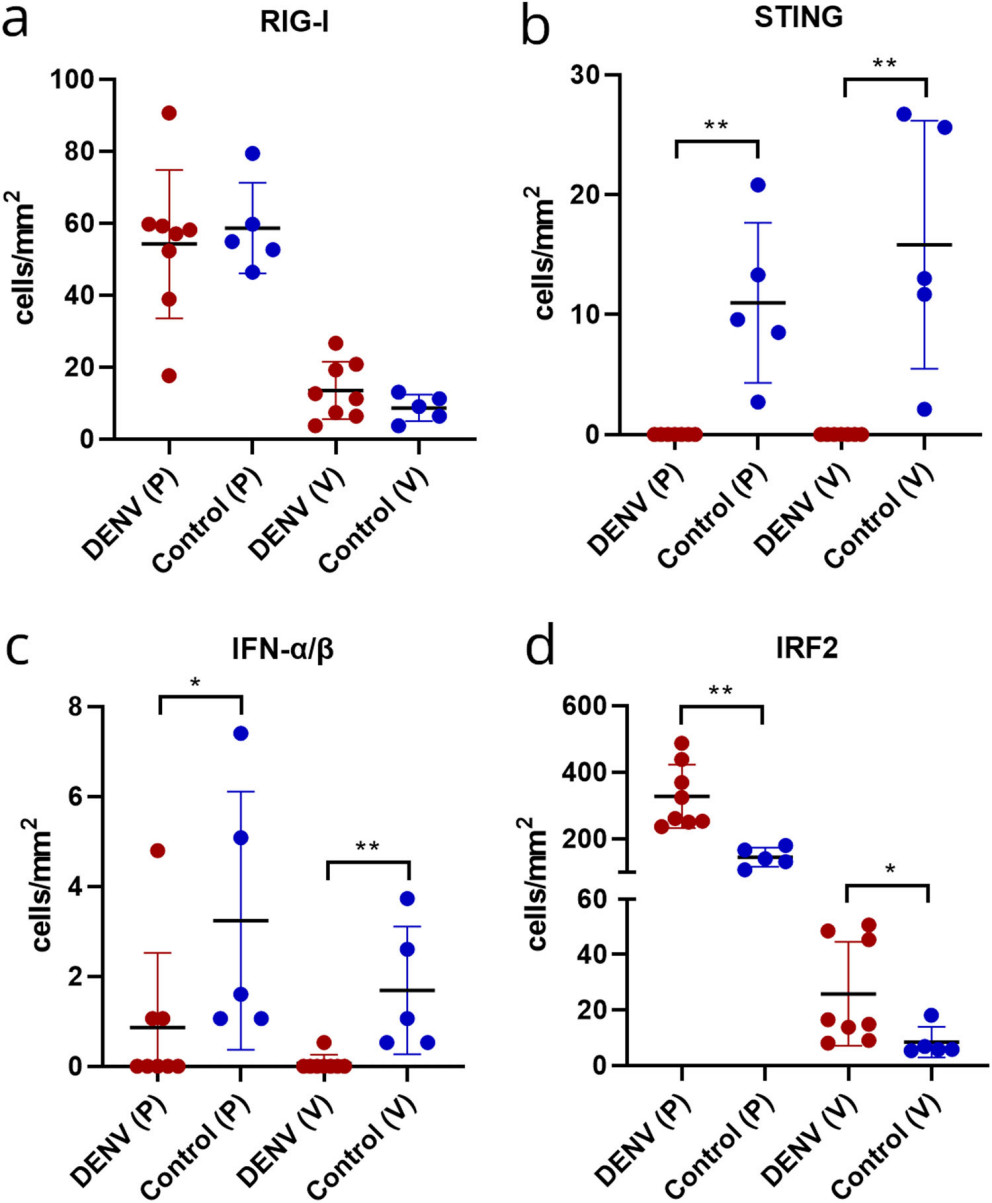
Graphical representation of the number of immunopositive cells in the cerebral cortex of severe dengue patients compared to control group. (a) RIG‐I, (b) STING, (c) IFN‐α/β, and (d) IRF2. Data were analyzed using the Mann–Whitney test. *p < 0.05; **p < 0.001. P: parenchyma; V: perivascular region.

Additionally, the innate immunity was quantitatively assessed by immunohistochemistry through the investigation of the following molecules: RIG‐I, STING, IFN‐α/β and IRF2 (Figure 5a–d).

**Figure 6:**
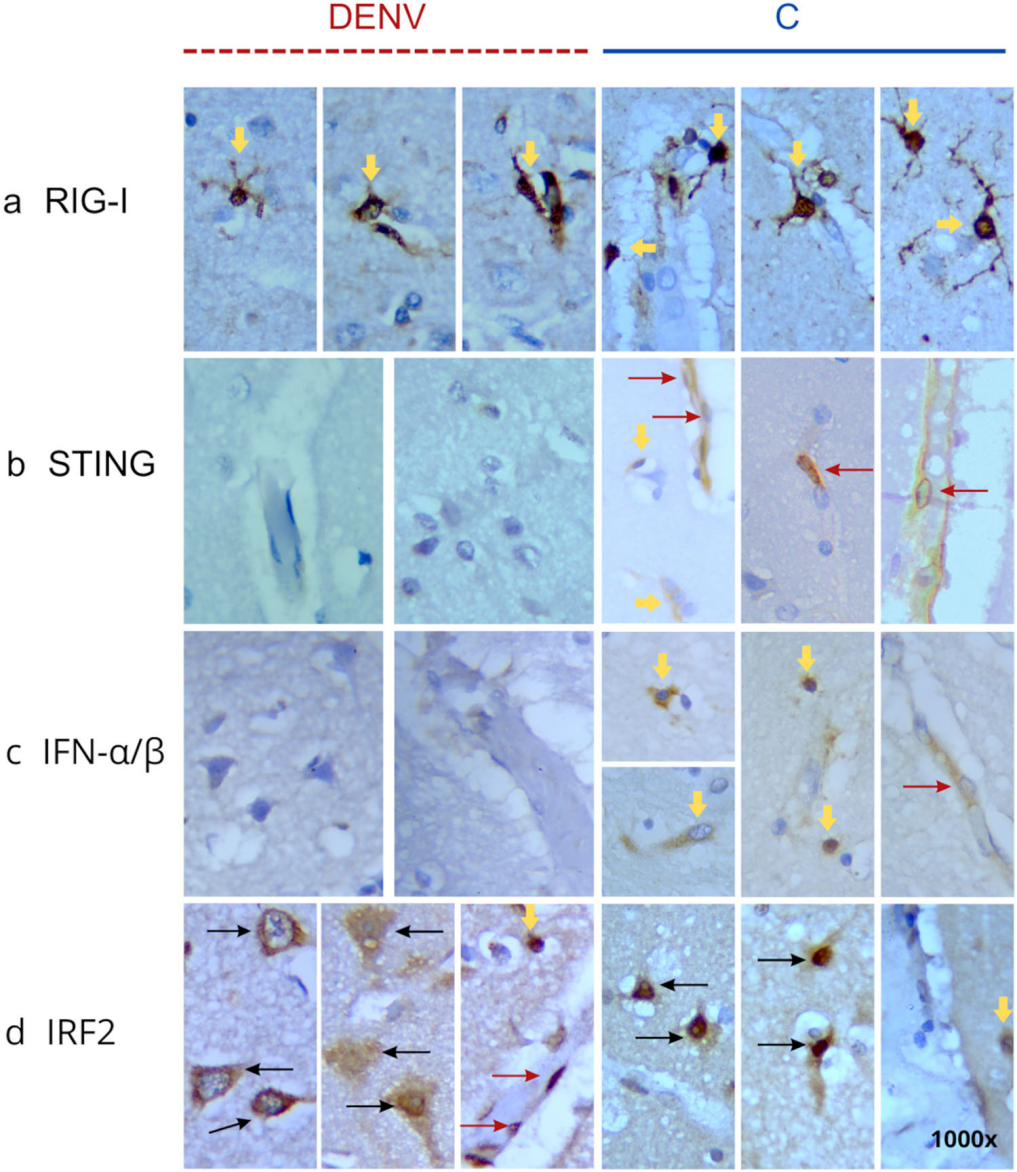
Representative images of immunolabeling in the cerebral cortex of patients with severe dengue and the control group. The panel shows brown‐stained immunopositive cells for RIG‐I (a), STING (b), IFN‐α/β (c), and IRF2 (d). Images on the left, outlined with a dark red dashed line, correspond to DENV cases; images on the right, outlined with a solid blue line, represent control cases. Dark red arrows: endothelial cells; yellow arrows: glial cells; black arrows: neuronal cell bodies. Immunoperoxidase reaction with Harris hematoxylin counterstaining; 1000× magnification.

RIG‐I expression was slightly in the perivascular region and moderate in the parenchyma, with the latter showing staining in neuroglial cells. There was no statistical difference in the number of stained cells in DENV cases, both in the parenchyma (54.18 cells/mm²; SD ± 20.12) and in the perivascular region (13.49 cells/mm²; SD ± 7.98), when compared to the respective regions in the control group (58.60 cells/mm², SD ± 12.57; 8.6 cells/mm², SD ± 3.7) (Figures 5a and 6a).

As for STING, no staining was observed in DENV cases, whereas in the control group, slight expression was noted in the parenchyma (10.98 cells/mm², SD ± 6.67), possibly in neuroglial cells, as well as in endothelial cells and the perivascular region (15.82 cells/mm², SD ± 10.33). The difference between the experimental and control groups was significant (*p* = 0.0013, Figures 5b and 6b).

IFN‐α/β was observed in only three DENV cases, but at low levels. Expression in the control group was also low, both in the parenchyma (3.24 cells/mm², SD ± 2.87) and in the perivascular region (1.69 cells/mm², SD ± 2.87). However, there was a statistically significant difference between the DENV and control groups (parenchyma, *p* = 0.0287; perivascular region, *p* = 0.0023; Figures 5c and 6c).

There was a pronounced increase in IRF2 in DENV cases, both in the parenchyma (327.2 cells/mm², SD ± 95.84) and the perivascular area (25.83 cells/mm², SD ± 18.74), with intense staining in neurons, neuroglial, and endothelial cells (Figure 6d) compared to the control group (145 cells/mm², SD ± 28.77; 8.3 cells/mm², SD ± 5.4). The observed increase in IRF2 was statistically significant (parenchyma, *p* = 0.0016; perivascular region, *p* = 0.0287; Figures 5d and 6d).

## 4 Discussion

The main findings of this study provide novel insights into dengue virus (DENV) involvement in the human central nervous system, specifically within the cerebral cortex of patients with severe dengue. Significant histopathological alterations were observed, including neuronal morphological changes and evidence of tissue damage. Notably, DENV antigen was detected by immunohistochemistry in both parenchymal and meningeal blood vessels, predominantly in cells with morphological features consistent with endothelial cells. In addition, our results also indicate a dysregulation of type I interferon signaling pathways, as evidenced by the altered expression of selected innate immune markers. These aspects are further discussed below.

### 4.1 Histopathological Features

Our histopathological analyses of the cerebral cortex from eight patients who died with a diagnosis of dengue revealed consistent cellular alterations across multiple samples. The most recurrent findings included neurons with contracted cytoplasm and pyknotic nuclei, central chromatolysis, neuronal loss, reactive gliosis, and demyelination of the white matter. These alterations are consistent with those reported by Salomão et al. [28], who described similar changes in the cerebral cortex of three fatal dengue cases. Likewise, Rivera et al. [39] identified comparable histopathological features in the brains of 16 out of 95 fatal dengue cases, with edema noted as the most frequent macroscopic alteration. In our series, one patient presented with intense hemorrhage in the meningeal region, while others exhibited marked vascular congestion within the parenchyma, findings that have also been previously documented in brain tissues from DHF patients [28, 39, 40].

### 4.2 Viremia in the Cerebral Cortex

One of the most significant findings was the evidence of substantial viremia, with the detection of the DENV antigen in vascular endothelial cells and mononuclear cells within the cortical parenchyma and meninge. Flaviviruses may access the central nervous system (CNS) through several distinct mechanisms, including: (a) infection of brain microvascular endothelial cells, leading to disruption of tight junctions and increased blood–brain barrier (BBB) permeability; (b) the “Trojan horse” strategy, in which infected monocytes cross the BBB and induce inflammation through cytokine release and endothelial activation; (c) invasion via the choroid plexus and cerebrospinal fluid; and (d) entry through peripheral nerves by retrograde axonal transport [41, 42, 43].

In the context of Dengue virus (DENV) infection, studies have demonstrated the virus's ability to infect and disrupt endothelial cells, which may contribute to the breakdown of the blood–brain barrier (BBB) and facilitate viral entry into the central nervous system (CNS). Notably, Dalrymple and Mackow [44] showed that productive infection of human endothelial cells by DENV is mediated through interactions with heparan sulfate‐containing proteoglycans [44]. Furthermore, this susceptibility has been demonstrated in vitro for DENV‐2 in human umbilical vein endothelial cells (HUVECs) and for DENV‐4 in mouse brain endothelial cells (MBECs), where infection led to changes in tight junction integrity and increased permeability [45, 46].

On the other hand, in our study, a similar cytoplasmic staining pattern to that observed in the previously described cells was not detected in neuronal cell bodies [28]. The capacity for neuroinvasion, neurotropism, neurovirulence, and the clinical symptoms characteristic of viral encephalitis, have been better understood for flaviviruses such as the Japanese encephalitis virus (JEV), West Nile virus (WNV), and tick‐borne encephalitis virus [47, 48, 49, 50]. Dengue virus is generally considered non‐neurotropic virus. However, the dynamics and impact on CNS appear to be related to the degree of invasion in this tissue, as demonstrated in vivo and in vitro research [19, 51, 52].

### 4.3 Dysregulation of Type I Interferon Signaling

Regarding molecules related to innate immunity, these patients who died from dengue showed in the cerebral cortex unchanged expression of RIG‐I, along with suppression of STING (Stimulator of Interferon Gene) and IFN‐α/β compared to the control group. Recent studies have shown that flaviviruses use different strategies to evade the host response, particularly in the innate immunity [53, 54]. Proteins encoded by the DENV genome, known as nonstructural (NS), could interfere with different macromolecules in signaling pathways related to the antiviral response, mainly those mediated by IFN [54, 55].

Chazal and colleagues showed that RIG‐I binds to the highly structured and conserved 5' region of DENV and Zika nascent transcripts before capping and that this mechanism leads to interferon secretion by infected cells, inhibiting viral replication and activating immune cells, including macrophages [56]. However, DENV has camouflage mechanisms through the modification of viral RNAs [57], as well as the formation of subgenomic flavivirus RNA (sfRNA) resistant to degradation [58]. The accumulation of these sfRNAs prevented the activation of RIG‐I and its interaction with MAVS (mitochondrial antiviral‐signaling protein), an adapter protein located in the inner mitochondrial membrane [59, 60]. Furthermore, it has been shown that the NS3 protein of DENV could prevent the translocation of RIG‐I to MAVS by interacting with the chaperone protein 14‐3‐3ϵ [61]. The interaction between RIG‐I and MAVS can also be inhibited by NS4a through its binding to the N‐terminal CARD‐like domain and the C‐terminal transmembrane domain of MAVS [62]. Furthermore, the proteins NS2a and NS4b from different DENV serotypes were able to abolish IFN‐β production by blocking the RIG‐I/MAVS pathway and inhibiting phosphorylation of TBK1/IRF [63].

In this study, STING, a protein essential for the innate immune response and primarily activated by intermediate viral RNA molecules, was not detected in the cerebral cortex of DENV‐positive cases. Once activated, STING leads to the phosphorylation and activation of the transcription factor IRF3 (Interferon Regulatory Factor 3) through the kinase TBK1 (TANK‐binding kinase 1), resulting in the transcription and production of type I interferons (IFN‐I), particularly IFN‐β, and pro‐inflammatory cytokines [64, 65]. STING can also activate the nuclear factor kappa B (NF‐κB) pathway, contributing to the production of inflammatory mediators [66]. Nonetheless, it has been shown that the complex protease DENV NSB3 can degrade STING, leading to the inhibition of type I Interferon production in human monocyte‐derived dendritic cells (MDDCs), as well as reversing this effect when mutated versions of STING were used, confirming this molecule as an important target for DENV circumvention strategy [4, 67, 68].

Thus, the action of DENV NS proteins on RIG‐I and STING, as previously explained, interferes with the production of IFN‐α/β, that may explain the reduced levels of these cytokines observed in our study. IFN‐α/β binds to their specific receptors, IFN‐α/β (IFNAR), triggering the activation of the JAK/STAT pathway through the phosphorylation of the adapter molecules TYK2 and JAK1 [69]. This leads to the phosphorylation and dimerization of various signal transduction molecules and the activation of transcription factors (STATs), such as STAT1 and STAT2. The phosphorylated STATs then form a heterotrimeric complex with IRF9, known as the IFN‐stimulated gene factor 3 (ISGF3), which translocates to the nucleus and activates ISGs to fight the virus [7, 70, 71].

In addition, in the cerebral cortex region, we observed an increase in IRF2 expression in neuronal, glial, and endothelial cells, a transcription factor constitutively expressed. IRF2 has primarily been described as a negative regulator of IFN signaling, antagonistically competing with IRF1 for binding to the same promoter elements or IFN‐inducing genes [72, 73, 74]. However, Li and collaborators, using a knockout mouse model for IRF2, demonstrated that this regulator plays a key role in protecting against the neuroinvasion of Sindbis virus when injected intraperitoneally [75]. Furthermore, it was recently shown the importance of IRF2 in the double‐stranded RNA‐induced antiviral pathway driven by OAS‐RNase L, which detects and degrades viral RNAs. IRF2 was identified as a key regulator of OAS3 and the rapid activation of the RNase L endonuclease [76].

Altogether, our results from cerebral cortex samples of patients with dengue who died to the disease suggest the ability of dengue virus to significantly infect endothelial cells, a key component of the blood‐brain barrier, causing neuroinflammation and characteristic cellular damage in nervous tissue. Additionally, we observed an in‐situ reduction in the expression of molecules related to innate immunity, particularly in the type I interferon signaling pathway, such as STING and IFN‐α/β, corroborating findings from experimental models regarding the evasion mechanisms employed by the virus. The upregulation of IRF2 observed in our study suggests a potential involvement in the local immune response within the CNS during severe dengue. However, whether this modulation exerts a protective or pathogenic effect remains to be elucidated. Further functional studies are needed to clarify the biological role of IRF2 in the context of dengue‐associated neuropathogenesis.

### 4.4 Study Limitations and Considerations Regarding Post‐Mortem Samples

Our study is based on post‐mortem human CNS tissue, which represents a significant strength in terms of clinical relevance, particularly in comparison to experimental animal models that may not fully recapitulate human dengue neuropathogenesis. However, we recognize several important limitations associated with this type of material.

First, post‐mortem studies are inherently descriptive and semi‐quantitative, with functional validation often limited by tissue preservation. As they represent a single time point, they may fail to capture transient molecular events such as phosphorylation and ubiquitination. Although we detected no changes in total RIG‐I protein levels (Figures 5a and 6a), we could not assess its activation status, which would require detection of posttranslational modifications using fresh samples or cell‐based systems. Second, the sample representativeness is limited, making it difficult to determine whether observed findings are specific to severe dengue or also present in milder forms. Third, the possible influence of comorbidities cannot be ruled out. Despite efforts to review available clinical records, premortem data were often incomplete, and undiagnosed conditions may have impacted the histopathological and immunological features observed.

Despite these challenges, we believe that the findings from post‐mortem samples remain highly valuable. They provide a rare opportunity to explore human neuroimmune responses to dengue virus in situ, which is particularly important given the current knowledge gaps in dengue‐related neuropathology.

## Author Contributions

All authors contributed to the study's conception and design. Data collection was performed by L.M.C., E.R.F., and C.P. Material preparation and analysis were performed by L.M.C., E.R.F., J.A.S.Q., C.L.P.L., and C.P. The first draft of the manuscript was written by L.M.C. and C.P.; M.I.S.D., L.V.C., R.P., P. F. C.V., E.S.M., and M.N.S. commented on previous versions of the manuscript. All authors read and approved of the final manuscript.

## Ethics Statement

The research was approved by the Ethics and Research Committee of Medical School, University of Sao Paulo (Protocol number: 253/12), in accordance with the Brazilian National Health Council (through Resolution number: 466/12.

## Conflicts of Interest

The authors declare no conflicts of interest.

## Data Availability

The data that support the findings of this study are available from the corresponding author upon reasonable request.

## References

[1] Z. Zeng , J. Zhan , L. Chen , H. Chen , and S. Cheng , “Global, Regional, and National Dengue Burden From 1990 to 2017: A Systematic Analysis Based on the Global Burden of Disease Study 2017,” EClinicalMedicine 32 (2021): 100712, 10.1016/j.eclinm.2020.100712. 33681736 PMC7910667

[2] D. C. Andrioli , M. A. Busato , and J. A. Lutinski , “Spatial and Temporal Distribution of Dengue in Brazil, 1990–2017,” PLoS One 15 (2020): e0228346, 10.1371/journal.pone.0228346. 32053623 PMC7018131

[3] R. M. Oneda , S. R. Basso , L. R. Frasson , N. M. Mottecy , L. Saraiva , and C. Bassani , “Epidemiological Profile of Dengue in Brazil Between the Years 2014 and 2019,” Revista da Associação Médica Brasileira 67 (2021): 731–735, 10.1590/1806-9282.20210121. 34550264

[4] S. Sinha , K. Singh , Y. S. Ravi Kumar , et al., “Dengue Virus Pathogenesis and Host Molecular Machineries,” Journal of Biomedical Science 31 (2024): 43, 10.1186/s12929-024-01030-9. 38649998 PMC11036733

[5] W. H. Wang , A. N. Urbina , M. R. Chang , et al., “Dengue Hemorrhagic Fever—A Systemic Literature Review of Current Perspectives on Pathogenesis, Prevention and Control,” Journal of Microbiology, Immunology and Infection 53 (2020): 963–978, 10.1016/j.jmii.2020.03.007. 32265181

[6] P. Bhatt , S. P. Sabeena , M. Varma , and G. Arunkumar , “Current Understanding of the Pathogenesis of Dengue Virus Infection,” Current Microbiology 78 (2021): 17–32, 10.1007/s00284-020-02284-w. 33231723 PMC7815537

[7] N. Uno and T. M. Ross , “Dengue Virus and the Host Innate Immune Response,” Emerging Microbes & Infections 7 (2018): 1–11, 10.1038/s41426-018-0168-0. 30301880 PMC6177401

[8] V. V. Costa , C. T. Fagundes , D. F. Valadão , et al., “Subversion of Early Innate Antiviral Responses During Antibody‐Dependent Enhancement of Dengue Virus Infection Induces Severe Disease in Immunocompetent Mice,” Medical Microbiology and Immunology 203 (2014): 231–250, 10.1007/s00430-014-0334-5. 24723052

[9] M. G. Guzman , M. Alvarez , and S. B. Halstead , “Secondary Infection as a Risk Factor for Dengue Hemorrhagic Fever/Dengue Shock Syndrome: An Historical Perspective and Role of Antibody‐Dependent Enhancement of Infection,” Archives of Virology 158 (2013): 1445–1459, 10.1007/s00705-013-1645-3. 23471635

[10] E. Z. Ong , S. L. Zhang , H. C. Tan , E. S. Gan , K. R. Chan , and E. E. Ooi , “Dengue Virus Compartmentalization During Antibody‐Enhanced Infection,” Scientific Reports 7 (2017): 40923, 10.1038/srep40923. 28084461 PMC5234037

[11] F. A. Bozza , O. G. Cruz , S. M. Zagne , et al., “Multiplex Cytokine Profile From Dengue Patients: MIP‐1beta and IFN‐Gamma as Predictive Factors for Severity,” BMC Infectious Diseases 8 (2008): 86, 10.1186/1471-2334-8-86. 18578883 PMC2474613

[12] E. L. Braga , P. Moura , L. M. Pinto , et al., “Detection of Circulant Tumor Necrosis Factor‐Alpha, Soluble Tumor Necrosis Factor p75 and Interferon‐Gamma in Brazilian Patients With Dengue Fever and Dengue Hemorrhagic Fever,” Memórias do Instituto Oswaldo Cruz 96 (2001): 229–232, 10.1590/S0074-02762001000200015. 11285501

[13] A. L. Rothman , “Cellular Immunology of Sequential Dengue Virus Infection and Its Role in Disease Pathogenesis,” Current Topics in Microbiology and Immunology 338 (2009): 83–98, 10.1007/978-3-642-02215-9-7. 19802580

[14] M. S. Coutinho‐da‐Silva , P. H. F. Sucupira , K. A. Bicalho , et al., “Serum Soluble Mediator Profiles and Networks During Acute Infection With Distinct DENV Serotypes,” Frontiers in immunology 13 (2022): e892990, 10.3389/fimmu.2022.892990. PMC9193801 35711447

[15] P. Henrique Ferreira Sucupira , M. Silveira Ferreira , M. Santos Coutinho‐da‐Silva , et al., “Serotype‐Associated Immune Response and Network Immunoclusters in Children and Adults During Acute Dengue Virus Infection,” Cytokine 169 (2023): 156306, 10.1016/j.cyto.2023.156306. 37542834

[16] U. K. Misra , J. Kalita , U. K. Syam , and T. N. Dhole , “Neurological Manifestations of Dengue Virus Infection,” Journal of the Neurological Sciences 244 (2006): 117–122, 10.1016/j.jns.2006.01.011. 16524594

[17] S. Trivedi and A. Chakravarty , “Neurological Complications of Dengue Fever,” Current Neurology and Neuroscience Reports 22 (2022): 515–529, 10.1007/s11910-022-01213-7. 35727463 PMC9210046

[18] V. Rangankar , D. Kumar , R. Kuber , and T. Kalekar , “Imaging of the Neurological Manifestations of Dengue: A Case Series,” South African Journal of Radiology 26 (2022): 1–10, 10.4102/sajr.v26i1.2528. PMC9724064 36483671

[19] J. H. Amorim , R. S. Pereira Bizerra , R. P. dos Santos Alves , et al., “A Genetic and Pathologic Study of a DENV2 Clinical Isolate Capable of Inducing Encephalitis and Hematological Disturbances in Immunocompetent Mice,” PLoS One 7 (2012): e44984, 10.1371/journal.pone.0044984. 23028722 PMC3441697

[20] M. Escudero‐Flórez , D. Torres‐Hoyos , Y. Miranda‐Brand , R. L. Boudreau , J. Gallego‐Gómez , and M. Vicente‐Manzanares , “Dengue Virus Infection Alters Inter‐Endothelial Junctions and Promotes Endothelial–Mesenchymal‐Transition‐Like Changes in Human Microvascular Endothelial Cells,” Viruses 15 (2023): 1437, 10.3390/v15071437. 37515125 PMC10386726

[21] M. E. H. Kayesh and K. Tsukiyama‐Kohara , “Mammalian Animal Models for Dengue Virus Infection: A Recent Overview,” Archives of Virology 167 (2022): 31–44, 10.1007/s00705-021-05298-2. 34761286 PMC8579898

[22] D. R. Palmer , P. Sun , C. Celluzzi , et al., “Differential Effects of Dengue Virus on Infected and Bystander Dendritic Cells,” Journal of Virology 79 (2005): 2432–2439, 10.1128/jvi.79.4.2432-2439.2005. 15681444 PMC546567

[23] M. L. Velandia‐Romero , M. A. Calderón‐Peláez , and J. E. Castellanos , “In Vitro Infection With Dengue Virus Induces Changes in the Structure and Function of the Mouse Brain Endothelium,” PLoS One 11 (2016): e0157786, 10.1371/journal.pone.0157786. 27336851 PMC4919088

[24] M. A. Calderón‐Peláez , M. L. Velandia‐Romero , L. Y. Bastidas‐Legarda , E. O. Beltrán , S. J. Camacho‐Ortega , and J. E. Castellanos , “Dengue Virus Infection of Blood‐Brain Barrier Cells: Consequences of Severe Disease,” Frontiers in Microbiology 10 (2019): e01435, 10.3389/fmicb.2019.01435. PMC6606788 31293558

[25] M. Puccioni‐Sohler and C. Rosadas , “Advances and New Insights in the Neuropathogenesis of Dengue Infection,” Arquivos de Neuro‐psiquiatria 73 (2015): 698–703, 10.1590/0004-282X20150074. 26222363

[26] N. G. Salomão , K. Rabelo , T. F. Póvoa , et al., “BALB/c Mice Infected With DENV‐2 Strain 66985 by the Intravenous Route Display Injury in the Central Nervous System,” Scientific Reports 8 (2018): 9754, 10.1038/s41598-018-28137-y. 29950590 PMC6021404

[27] T. J. Shen , M. K. Jhan , J. C. Kao , et al., “A Murine Model of Dengue Virus‐Induced Acute Viral Encephalitis‐Like Disease,” Journal of Visualized Experiments 2019 (2019): e59132, 10.3791/59132. 31081826

[28] N. Salomão , K. Rabelo , C. Basílio‐De‐Oliveira , et al., “Fatal Dengue Cases Reveal Brain Injury and Viral Replication in Brain‐Resident Cells Associated With the Local Production of Pro‐Inflammatory Mediators,” Viruses 12 (2020): 603, 10.3390/v12060603. 32486462 PMC7354550

[29] A. E. Ngono and S. Shresta , “Immune Response to Dengue and Zika,” Annual Review of Immunology 36 (2018): 279–308, 10.1146/annurev-immunol-042617-053142. PMC5910217 29345964

[30] A. M. A. Nasirudeen , H. H. Wong , P. Thien , S. Xu , K. P. Lam , and D. X. Liu , “RIG‐i, MDA5 and TLR3 Synergistically Play an Important Role in Restriction of Dengue Virus Infection,” PLoS Neglected Tropical Diseases 5 (2011): e926, 10.1371/journal.pntd.0000926. 21245912 PMC3014945

[31] S. K. Roy and S. Bhattacharjee , “Dengue Virus: Epidemiology, Biology, and Disease Aetiology,” Canadian Journal of Microbiology 67 (2021): 687–702, 10.1139/cjm-2020-0572. 34171205

[32] H. Ishikawa , Z. Ma , and G. N. Barber , “STING Regulates Intracellular DNA‐Mediated, Type i Interferon‐Dependent Innate Immunity,” Nature 461 (2009): 788–792, 10.1038/nature08476. 19776740 PMC4664154

[33] J. Tao , X. Zhou , and Z. Jiang , “Cgas‐Cgamp‐Sting: The Three Musketeers of Cytosolic DNA Sensing and Signaling,” IUBMB Life 68 (2016): 858–870, 10.1002/iub.1566. 27706894

[34] A. C. Stabell , N. R. Meyerson , R. C. Gullberg , et al., “Dengue Viruses Cleave Sting in Humans But Not in Nonhuman Primates, Their Presumed Natural Reservoir,” eLife 7 (2018): e31919, 10.7554/eLife.31919. 29557779 PMC5860865

[35] L. G. Webb and A. Fernandez‐Sesma , “RNA Viruses and the cGAS‐Sting Pathway: Reframing Our Understanding of Innate Immune Sensing,” Current Opinion in Virology 53 (2022): 101206, 10.1016/j.coviro.2022.101206. 35180533

[36] O. V. Matveeva and P. M. Chumakov , “Defects in Interferon Pathways as Potential Biomarkers of Sensitivity to Oncolytic Viruses,” Reviews in Medical Virology 28 (2018): e2008, 10.1002/rmv.2008. 30209859 PMC6906582

[37] T. Taniguchi , K. Ogasawara , A. Takaoka , and N. Tanaka , “IRF Family of Transcription Factors as Regulators of Host Defense,” Annual Review of Immunology 19 (2001): 623–655, 10.1146/annurev.immunol.19.1.623. 11244049

[38] M. Á. García‐Cabezas , Y. J. John , H. Barbas , and B. Zikopoulos , “Distinction of Neurons, Glia and Endothelial Cells in the Cerebral Cortex: An Algorithm Based on Cytological Features,” Frontiers in Neuroanatomy 10 (2016): e00107, 10.3389/fnana.2016.00107. PMC5088408 27847469

[39] J. A. Rivera , A. C. Rengifo , E. A. Parra , J. E. Castellanos , and M. L. Caldas , “Illustrated Histopathological Features of Fatal Dengue Cases in Colombia,” Biomedica: Revista del Instituto Nacional de Salud 40 (2020): 438–447, 10.7705/biomedica.5016. 33030821 PMC7666849

[40] K. A. P. Idirisinghe , “Histopathological Study of Dengue Haemorrhagic Fever,” Journal of Diagnostic Pathology 8 (2014): 50, 10.4038/jdp.v8i1.6790.

[41] Y. M. Mustafá , L. M. Meuren , S. V. A. Coelho , and L. B. De Arruda , “Pathways Exploited by Flaviviruses to Counteract the Blood‐Brain Barrier and Invade the Central Nervous System,” Frontiers in Microbiology 10 (2019): 525, 10.3389/fmicb.2019.00525. 30984122 PMC6447710

[42] J. W. Neal , “Flaviviruses Are Neurotropic, but How Do They Invade the CNS?,” Journal of Infection 69 (2014): 203–215, 10.1016/j.jinf.2014.05.010. 24880028

[43] L. de Vries and A. T. Harding , “Mechanisms of Neuroinvasion and Neuropathogenesis by Pathologic Flaviviruses,” Viruses 15 (2023): 261, 10.3390/v15020261. 36851477 PMC9965671

[44] N. Dalrymple and E. R. Mackow , “Productive Dengue Virus Infection of Human Endothelial Cells Is Directed by Heparan Sulfate‐Containing Proteoglycan Receptors,” Journal of Virology 85 (2011): 9478–9485, 10.1128/jvi.05008-11. 21734047 PMC3165770

[45] P. Avirutnan , P. Malasit , B. Seliger , S. Bhakdi , and M. Husmann , “Dengue Virus Infection of Human Endothelial Cells Leads to Chemokine Production, Complement Activation, and Apoptosis,” Journal of Immunology 161 (1998): 6338–6346, 10.4049/jimmunol.161.11.6338. 9834124

[46] M. L. Velandia‐Romero , O. Acosta‐Losada , and J. E. Castellanos , “In Vivo Infection by a Neuroinvasive Neurovirulent Dengue Virus,” Journal of Neurovirology 18 (2012): 374–387, 10.1007/s13365-012-0117-y. 22825914

[47] E. Gould and T. Solomon , “Pathogenic Flaviviruses,” Lancet 371 (2008): 500–509, 10.1016/S0140-6736(08)60238-X. 18262042

[48] R. Lindqvist , A. Upadhyay , and A. K. Överby , “Tick‐Borne Flaviviruses and the Type I Interferon Response,” Viruses 10 (2018): 340, 10.3390/v10070340. 29933625 PMC6071234

[49] O. A. Maximova and A. G. Pletnev , “Flaviviruses and the Central Nervous System: Revisiting Neuropathological Concepts,” Annual Review of Virology 5 (2018): 255–272, 10.1146/annurev-virology-092917-043439. 30265628

[50] G. J. Sips , J. Wilschut , and J. M. Smit , “Neuroinvasive Flavivirus Infections,” Reviews in Medical Virology 22 (2012): 69–87, 10.1002/rmv.712. 22086854

[51] T. T. Tsai , C. L. Chen , C. C. Tsai , and C. F. Lin , “Targeting Heat Shock Factor 1 as an Antiviral Strategy Against Dengue Virus Replication In Vitro and In Vivo,” Antiviral Research 145 (2017): 44–53, 10.1016/j.antiviral.2017.07.008. 28733114

[52] A. Tuiskunen , M. Wahlström , J. Bergström , P. Buchy , I. Leparc‐Goffart , and Å. Lundkvist , “Phenotypic Characterization of Patient Dengue Virus Isolates in BALB/c Mice Differentiates Dengue Fever and Dengue Hemorrhagic Fever From Dengue Shock Syndrome,” Virology Journal 8 (2011): 398, 10.1186/1743-422X-8-398. 21835036 PMC3170302

[53] A. M. Green , P. R. Beatty , A. Hadjilaou , and E. Harris , “Innate Immunity to Dengue Virus Infection and Subversion of Antiviral Responses,” Journal of Molecular Biology 426 (2014): 1148–1160, 10.1016/j.jmb.2013.11.023. 24316047 PMC4174300

[54] M. F. Lee , G. Z. Voon , H. X. Lim , M. L. Chua , and C. L. Poh , “Innate and Adaptive Immune Evasion by Dengue Virus,” Frontiers in Cellular and Infection Microbiology 12 (2022): e1004608, 10.3389/fcimb.2022.1004608. PMC9523788 36189361

[55] A. Tuiskunen Bäck and Å. Lundkvist , “Dengue Viruses—An Overview,” Infection Ecology & Epidemiology 3 (2013): e19839, 10.3402/iee.v3i0.19839. PMC3759171 24003364

[56] M. Chazal , G. Beauclair , S. Gracias , et al., “RIG‐I Recognizes the 5′ Region of Dengue and Zika Virus Genomes,” Cell Reports 24 (2018): 320–328, 10.1016/j.celrep.2018.06.047. 29996094

[57] S. Potisopon , S. Priet , A. Collet , E. Decroly , B. Canard , and B. Selisko , “The Methyltransferase Domain of Dengue Virus Protein NS5 Ensures Efficient RNA Synthesis Initiation and Elongation by the Polymerase Domain,” Nucleic Acids Research 42 (2014): 11642–11656, 10.1093/nar/gku666. 25209234 PMC4191377

[58] B. D. Clarke , J. A. Roby , A. Slonchak , and A. A. Khromykh , “Functional Non‐Coding Rnas Derived From the Flavivirus 3' Untranslated Region,” Virus Research 206 (2015): 53–61, 10.1016/j.virusres.2015.01.026. 25660582

[59] G. Manokaran , E. Finol , C. Wang , et al., “Dengue Subgenomic RNA Binds TRIM25 to Inhibit Interferon Expression for Epidemiological Fitness,” Science (New York, N.Y.) 350 (2015): 217–221, 10.1126/science.aab3369. 26138103 PMC4824004

[60] M. Okamoto , T. Kouwaki , Y. Fukushima , and H. Oshiumi , “Regulation of RIG‐I Activation by K63‐Linked Polyubiquitination,” Frontiers in Immunology 8 (2018): e01942, 10.3389/fimmu.2017.01942. PMC5760545 29354136

[61] Y. K. Chan and M. U. Gack , “A Phosphomimetic‐Based Mechanism of Dengue Virus to Antagonize Innate Immunity,” Nature Immunology 17 (2016): 523–530, 10.1038/ni.3393. 26998762 PMC4837045

[62] Z. He , X. Zhu , W. Wen , et al., “Dengue Virus Subverts Host Innate Immunity by Targeting Adaptor Protein MAVS,” Journal of Virology 90 (2016): 7219–7230, 10.1128/jvi.00221-16. 27252539 PMC4984625

[63] N. A. Dalrymple , V. Cimica , and E. R. Mackow , “Dengue Virus NS Proteins Inhibit RIG‐I/MAVS Signaling by Blocking TBK1/IRF3 Phosphorylation: Dengue Virus Serotype 1 NS4A Is a Unique Interferon‐Regulating Virulence Determinant,” mBio 6 (2015): e00553–15, 10.1128/mBio.00553-15. 25968648 PMC4436066

[64] G. N. Barber , “Sting‐Dependent Signaling,” Nature Immunology 12 (2011): 929–930, 10.1038/ni.2118. 21934672

[65] Y. Tanaka and Z. J. Chen , “STING Specifies IRF3 Phosphorylation by TBK1 in the Cytosolic DNA Signaling Pathway,” Science Signaling 5 (2012): ra20, 10.1126/scisignal.2002521. 22394562 PMC3549669

[66] S. Yum , M. Li , Y. Fang , and Z. J. Chen , “TBK1 Recruitment to STING Activates Both IRF3 and NF‐κB That Mediate Immune Defense Against Tumors and Viral Infections,” Proceedings of the National Academy of Sciences 118 (2021): e2100225118, 10.1073/pnas.2100225118. PMC8040795 33785602

[67] S. Aguirre , A. M. Maestre , S. Pagni , et al., “DENV Inhibits Type I IFN Production in Infected Cells by Cleaving Human STING,” PLoS Pathogens 8 (2012): e1002934, 10.1371/journal.ppat.1002934. 23055924 PMC3464218

[68] T. Zhu , L. G. Webb , J. Veloz , M. Wilkins , S. Aguirre , and A. Fernandez‐Sesma , “Generation and Characterization of Human‐Mouse STING Chimeras That Allow DENV Replication in Mouse Cells,” mSphere 7 (2022): e0091421, 10.1128/msphere.00914-21. 35477320 PMC9241525

[69] L. Velazquez , M. Fellous , G. R. Stark , and S. Pellegrini , “A Protein Tyrosine Kinase in the Interferon α β Signaling Pathway,” Cell 70 (1992): 313–322, 10.1016/0092-8674(92)90105-L. 1386289

[70] L. B. Ivashkiv and L. T. Donlin , “Regulation of Type i Interferon Responses,” Nature Reviews Immunology 14 (2014): 36–49, 10.1038/nri3581. PMC4084561 24362405

[71] L. C. Platanias , “Mechanisms of Type‐I‐ and Type‐II‐Interferon‐Mediated Signalling,” Nature Reviews Immunology 5 (2005): 375–386, 10.1038/nri1604. 15864272

[72] A. Antonczyk , B. Krist , M. Sajek , et al., “Direct Inhibition of IRF‐Dependent Transcriptional Regulatory Mechanisms Associated With Disease,” Frontiers in Immunology 10 (2019): 1176, 10.3389/fimmu.2019.01176. 31178872 PMC6543449

[73] H. Harada , T. Fujita , M. Miyamoto , et al., “Structurally Similar but Functionally Distinct Factors, IRF‐1 and IRF‐2, Bind to the Same Regulatory Elements of IFN and IFN‐Inducible Genes,” Cell 58 (1989): 729–739, 10.1016/0092-8674(89)90107-4. 2475256

[74] K. Minamino , K. Takahara , T. Adachi , et al., “IRF‐2 Regulates B‐Cell Proliferation and Antibody Production Through Distinct Mechanisms,” International Immunology 24 (2012): 573–581, 10.1093/intimm/dxs060. 22773153

[75] M. M. H. Li , L. Bozzacco , H. H. Hoffmann , et al., “Interferon Regulatory Factor 2 Protects Mice From Lethal Viral Neuroinvasion,” Journal of Experimental Medicine 213 (2016): 2931–2947, 10.1084/jem.20160303. 27899441 PMC5154937

[76] S. Oh , G. Santiago , L. Manjunath , et al., “A CRISPR‐Cas9 Knockout Screening Identifies IRF2 as a Key Driver of OAS3/RNase L‐Mediated RNA Decay During Viral Infection,” Proceedings of the National Academy of Sciences 121, no. 45. 10.1073/pnas.2412725121. PMC11551408 39475651

